# Immune Mechanisms and Translational Study Design in Viral Vaccine Development

**DOI:** 10.3390/ijms27135790

**Published:** 2026-06-26

**Authors:** Stephanie Lim, Byron Martina

**Affiliations:** 1 Artemis Bioservices, Molengraasssingel 10, 2629 JD Delft, The Netherlands; s.lim@artemisbioservices.com; 2 School of Medicine, The Avalon University, WTC, Piscaderaweg z/n, Willemstad, Curaçao; 3 Curacao Biomedical & Health Reseach Institute, Pater Euwensweg 36, Willemstad, Curaçao

**Keywords:** viral vaccine development, immune mechanisms, T cell immunity, antibody effector function, translational models, correlates of protection

## Abstract

Viral vaccine development requires both mechanistic understanding of protective immunity and translational study designs that connect preclinical data with human outcomes. Animal models remain important for early assessment of safety, immunogenicity and protective efficacy, but their predictive value depends on the question being asked, the pathophysiology of infection, the immune mechanisms expected to mediate protection, and the biomarkers chosen to bridge animal and human data. This review focuses on viral vaccines and examines innate and adaptive mechanisms of vaccine-induced protection, including B cell and antibody responses, Fc-mediated functions, Fc glycosylation, T cell memory and CD8+ cytotoxic responses. We discuss common reasons for clinical failure and show how preclinical endpoints can be classified as human-counterpart, surrogate or comparative/mechanistic readouts. Influenza and COVID-19 examples illustrate how different models can be combined across discovery, challenge, transmission and late-stage bridging studies. Emerging tools such as systems serology, omics, AI/ML and new approach methods can improve candidate prioritization, but their value depends on assay standardization, biological validation and cautious interpretation. A mechanism-driven model cascade, paired with human-relevant immunological readouts, can improve preclinical interpretation and reduce the risk of advancing candidates that are unlikely to succeed in clinical trials.

## 1. Introduction

Vaccines reduce morbidity and mortality caused by infectious diseases, including SARS-CoV-2, hepatitis viruses, human papillomavirus, polio and yellow fever [1,2]. The COVID-19 pandemic showed how rapidly vaccines may be needed once a new pathogen begins spreading in humans [3]. It also provided clear examples of accelerated clinical development, with mRNA and adenoviral-vectored vaccines showing efficacy in large phase 3 trials within the first year of the pandemic [4,5,6].

Despite these successes, vaccine development remains a staged process. It begins with discovery and candidate design, followed by preclinical testing and then phase 1, phase 2 and phase 3 clinical trials. Safety is central throughout the process because vaccines are often given to healthy people and may be intended for vulnerable groups, including young infants, older adults or immunocompromised individuals [2]. Preclinical studies are used to identify unacceptable toxicity, characterize immune responses, estimate protective potential and select candidates with a plausible path to clinical testing.

Animal models are a major part of this preclinical toolkit. Common models include mice and rats, while non-rodent models such as hamsters, guinea pigs, cotton rats, ferrets, rabbits, mini pigs and non-human primates may be used when they better reflect the biology of the target infection. The use of non-human primates is under increasing ethical and regulatory scrutiny, which reinforces the need to select models that answer a defined question rather than defaulting to the most complex species available [7].

Candidates that perform well in preclinical studies may progress to phase 1 and phase 2 clinical trials. Phase 1 studies assess safety and tolerability in a small group of healthy participants [5]. Phase 2 studies refine dose, schedule and immune readouts. These readouts often include neutralizing antibodies, and may also include T cell immunity, Fc-mediated antibody functions or other correlates of protection depending on the pathogen and vaccine platform [8,9,10].

The transition from animal studies to human trials remains a weak point. Animal models can identify mechanisms and de-risk candidates, but they do not always predict human vaccine efficacy with sufficient precision [11]. Drug and vaccine candidates may fail during late clinical development because of insufficient efficacy, safety concerns, endpoint mismatch or reliance on immune markers that are not validated for the target population or clinical endpoint [12,13].

Translational gaps can also arise from biological heterogeneity in the host and pathogen. Host genetic diversity, age, prior immune exposure and pathogen variability can alter vaccine responses, supporting complementary model systems, human natural history cohorts and high-dimensional immunoprofiling rather than reliance on a single average immune readout [14,15].

Between conventional preclinical studies and large field efficacy trials, controlled human infection models can sometimes provide a useful bridge. When ethically acceptable and technically feasible, these studies expose vaccinated volunteers to a defined pathogen challenge and can generate high-resolution data on early infection, safety and correlates of protection. They cannot replace broader efficacy studies and are appropriate only for selected pathogens, but they can reduce uncertainty when animal models or field trials cannot answer a mechanistic question efficiently [16,17,18].

This review focuses on viral vaccine development rather than attempting to cover bacterial or fungal vaccines comprehensively. It addresses three linked questions: which immune mechanisms support vaccine-induced protection, which antibody and cellular features shape that protection, and how translational models and biomarkers can be selected to improve prediction of human vaccine efficacy.

## 2. Vaccine-Induced Immune Response and Protection

**Figure 1:**
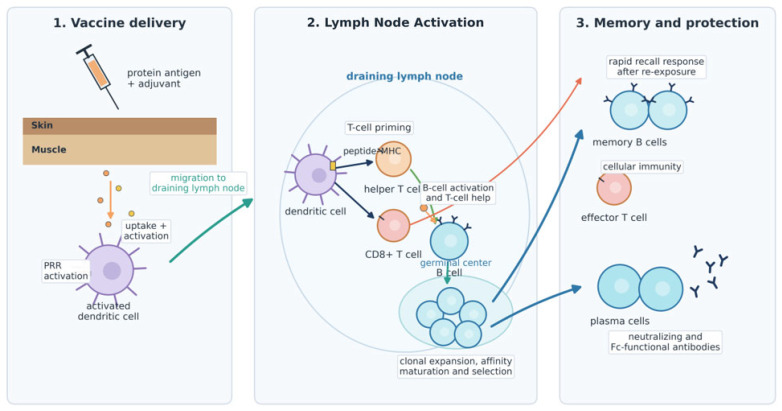
The immune response to vaccination with a conventional protein antigen (generated with ChatGPT, version 5.5). After intramuscular injection, the vaccine antigen and adjuvant are taken up by dendritic cells, which are activated through pattern-recognition receptors. Activated dendritic cells migrate to the draining lymph node and present antigen-derived peptides to T cells via major histocompatibility complex molecules. Helper T cells support B-cell activation, clonal expansion, affinity maturation, and class switching, leading to the formation of antibody-secreting plasma cells and memory B cells, while CD8+ T cells contribute to cellular effector responses [19]. Arrowheads indicate the direction of the illustrated processes: orange indicates antigen movement and uptake; teal indicates dendritic-cell migration to the draining lymph node; dark blue indicates peptide–MHC presentation and T-cell priming; green indicates helper-T-cell support of B cells; blue indicates germinal-center B-cell differentiation into memory B cells and plasma cells; and coral indicates progression of the CD8+ T-cell response toward cellular effector and recall protection.

Vaccines use the ability of the innate and adaptive immune system to recognize pathogenic antigens and generate immune memory. A conventional protein vaccine, for example, delivers an antigen with or without an adjuvant. Dendritic cells take up the antigen, respond to danger signals and move to draining lymph nodes, where they present antigen-derived peptides to T cells. Helper T cells then support B cell proliferation, affinity maturation, class switching and memory B cell formation (Figure 1) [19].

Adaptive immunity is mediated by B cells and T cells. B cells produce antibodies and drive humoral immunity, while T cells support cellular immunity by helping B cells, killing infected cells and shaping inflammatory responses. Most routinely used vaccines, with the exception of Bacillus Calmette–Guerin (BCG), are thought to protect mainly by inducing humoral immunity, although cellular immunity can still influence durability, breadth and protection against severe disease [20]. Recent human challenge data in orthoflavivirus vaccination further show that vaccine-induced T cell responses can contribute to control of acute viral infection even when neutralizing antibodies are absent or limited [21].

### Cellular Immunity and Long-Term Vaccine Protection

Cellular immunity is especially important when protection is not captured by neutralizing antibody titers alone. BCG is the classic example of a vaccine for which protection is associated largely with cellular and innate immune programming rather than a simple serum-antibody correlate [20]. More broadly, CD4+ T cells provide T follicular helper support for germinal-center responses and can also shape Th1 or Th17 immunity, whereas CD8+ cytotoxic T cells eliminate infected cells through perforin- and granzyme-mediated killing. Memory T cells, including circulating central and effector memory cells and tissue-resident memory cells at mucosal sites, can accelerate control of infection after re-exposure and may contribute to long-term protection even when circulating antibodies decline [20,21].

Preclinical studies should therefore include cellular immune readouts when the expected mechanism or clinical endpoint requires them. Useful measurements include antigen-specific CD4+ and CD8+ T cell frequency, cytokine polyfunctionality, cytotoxic markers, tissue-resident memory phenotypes and recall responses after infection challenge. These endpoints are particularly relevant when vaccines aim to prevent severe disease, reduce tissue pathology or maintain protection against antigenically shifted variants.

Key takeaway: humoral and cellular immunity should be treated as complementary layers of vaccine-induced protection; the relative emphasis should follow the pathogen, vaccine platform and intended clinical endpoint.

## 3. Antibodies as Correlates of Protection

The early humoral response is usually dominated by IgM. As the response matures, B cells undergo class switching and produce additional antibody isotypes, including IgG. Human IgG is divided into four subclasses: IgG1, IgG2, IgG3 and IgG4. Their approximate distribution in human serum is 60% IgG1, 32% IgG2, 4% IgG3 and 4% IgG4 [22].

All IgG subclasses can contribute to virus neutralization through the antigen-binding fragment (Fab). When the Fab domain binds viral epitopes involved in receptor binding, fusion or entry, it can prevent the virus from infecting host cells. Neutralizing antibodies are therefore an important correlate of protection for many vaccines [8,9]. In vaccine studies, neutralization is commonly measured with viral neutralization tests, while antigen-specific binding antibodies may be quantified with enzyme-linked immunosorbent assays.

Recent SARS-CoV-2 vaccine studies strengthened the evidence that antibody markers can predict protection at the population level. Neutralizing antibody levels were highly predictive of protection from symptomatic SARS-CoV-2 infection across vaccine platforms, and immune correlate analyses of the ChAdOx1 nCoV-19 and mRNA-1273 trials showed that higher binding and neutralizing antibody levels were associated with lower COVID-19 risk [23,24,25]. However, a single antibody titer does not explain all forms of protection, all variants, all tissues or all populations [13,26].

## 4. Key Takeaway: Neutralizing Antibody Titers Are Useful Bridging Markers, but They Should Be Interpreted with Cellular and Fc-Mediated Readouts When the Clinical Endpoint Includes Severe Disease, Mucosal Infection, Transmission or Antigenic Variants: Fc-Mediated Antibody Functions

Protection may also involve non-neutralizing or partly neutralizing antibody functions mediated by the fragment crystallizable (Fc) region. Antibodies bound to viral particles or infected cells can interact with complement proteins and Fc gamma receptors (FcγRs) on effector cells. Effector cells require a sufficient number of FcγRs to be engaged by immune complexes before activation occurs, a point often referred to as the activation threshold [27,28].

**Figure 2:**
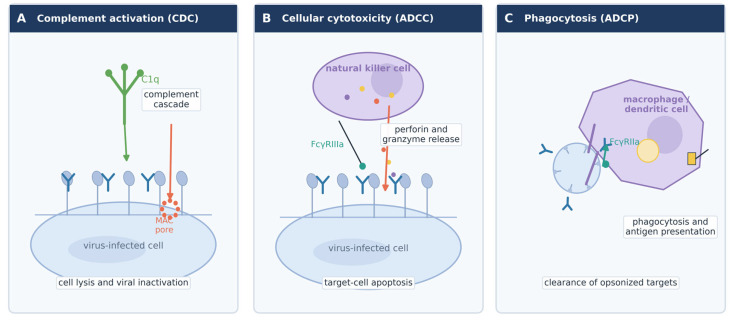
The contribution of Fc-mediated functions of IgG antibodies to vaccine-induced protection (adapted from [31]). (**A**) In complement-dependent cytotoxicity (CDC), C1q binds the Fc domains of antibodies on infected cells, initiates the complement cascade, and promotes membrane attack complex (MAC) formation and cell lysis. Complement-mediated membrane damage can also reduce the infectivity of viral particles. (**B**) In antibody-dependent cellular cytotoxicity (ADCC), natural killer cells are activated through FcγRIIIa binding to antibody Fc regions on infected cells, leading to the release of perforin and granzymes and apoptosis of the target cell [32]. (**C**) In antibody-dependent cellular phagocytosis (ADCP), FcγRIIa on macrophages and dendritic cells binds antibody Fc regions and promotes the uptake and clearance of antibody-coated targets, which can also support antigen presentation to T cells [33]. Arrowheads indicate the direction of the illustrated processes: the green arrow shows C1q-mediated initiation of complement activity, whereas the coral arrows show progression toward MAC formation or the directed release of cytotoxic granules. In panel C, the teal FcγRIIa symbol and purple contact lines indicate receptor-mediated capture and engulfment of the opsonized target. Dark-blue Y-shaped symbols represent IgG antibodies; light blue represents infected cells, opsonized targets, and their surface antigens; purple represents effector immune cells; green represents C1q; teal represents Fcγ receptors; and coral, orange, and yellow elements highlight downstream effector components, cytotoxic granules, or internalized and processed antigen.

Fc-mediated functions include antibody-dependent complement activation and cytotoxicity (CDC), antibody-dependent cellular cytotoxicity (ADCC) and antibody-dependent cellular phagocytosis (ADCP) (Figure 2). These mechanisms can contribute to protection by clearing infected cells, damaging viral particles, activating innate immune cells and shaping downstream adaptive immunity [29,30].

Recent vaccine studies show why Fc function should be measured selectively rather than inferred from binding antibody titer alone. COVID-19 vaccine platforms can induce distinct Fc effector profiles [34,35,36], and experimental SARS-CoV-2 challenge studies indicate that FcγR engagement can contribute to protection against antigenically shifted variants [37]. The translational point is not that every study requires a full systems serology panel, but that candidate mechanism and clinical endpoint should determine which antibody quality assays are included.

## 5. Antibody Quality Features Beyond Titer

Antibody-mediated protection has both quantitative and qualitative components. Titers indicate how much antibody is present, whereas specificity, affinity, avidity, subclass distribution, HCDR3 structure, hinge length, Fc glycosylation, Fc-receptor binding and persistence determine what that antibody can do [8,22,38]. The following sections separate antigen-binding features from Fc-dependent and durability features so that each readout can be linked to a defined mechanism and translational question.

## 6. HCDR3 and Antigen Binding

The efficacy of antibodies is closely linked to how they interact with their targets. Specificity, affinity and avidity all influence whether an antibody can bind the relevant epitope and remain bound long enough to block infection or mark the target for immune clearance. Beyond the affinity of a single Fab, avidity can increase through bivalent binding, antibody oligomerization and the formation of immune complexes that cross-link Fc-bearing cells, which helps explain why binding assays alone may not predict Fc effector potency [39]. One of the structural loops of the antibody variable region, the heavy chain complementarity determining region 3 (HCDR3), often has a dominant role in antigen-binding specificity.

HCDR3 length and composition can influence neutralizing capacity. Long or structurally extended HCDR3 loops may reach epitopes that are otherwise difficult to access because of glycosylation or recessed location on the pathogen surface. Such loops have been described in antibodies that neutralize or bind difficult targets, including HIV, malaria and African trypanosomes [40,41,42].

**Figure 3:**
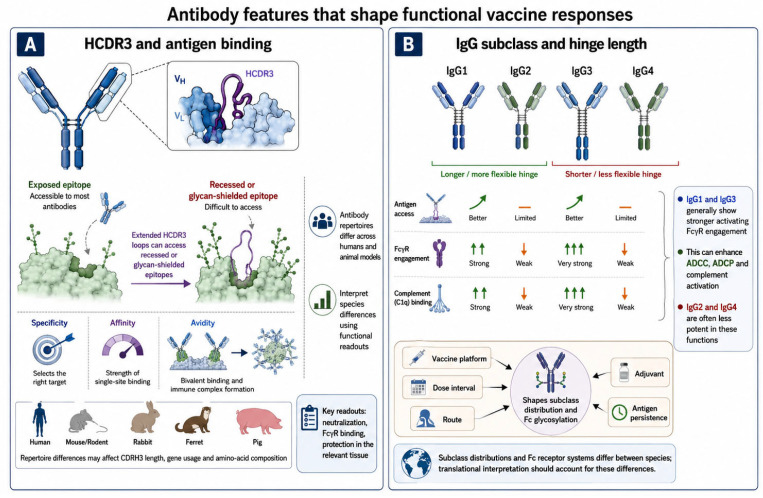
Antibody features that shape functional vaccine responses. (**A**) HCDR3-dependent antigen recognition. Specificity, affinity and avidity determine antibody binding and functional activity. Long or structurally extended HCDR3 loops may access recessed or glycan-shielded epitopes that are difficult to reach with conventional antibodies. Because antibody repertoires differ between humans and animal models, interpretation should be supported by functional readouts such as neutralization, FcγR binding and tissue protection. (**B**) IgG subclass and hinge length. IgG1 and IgG3 generally possess longer, more flexible hinges and stronger activating Fcγ receptor engagement than IgG2 and IgG4, resulting in enhanced ADCC, ADCP and complement activation. Vaccine platform, adjuvant, dose interval and antigen persistence can shape subclass distribution and Fc glycosylation. Species-specific Fc receptor biology should therefore be considered when translating animal model data to humans. AI (ChatGTP version 5.5) was used to generate the figure.

Species-specific antibody repertoires can affect how this feature is interpreted in animal models. Antibody repertoire studies show that CDRH3 length distributions, gene-segment usage and amino acid composition differ among humans, rodents, rabbits, ferrets and pigs rather than following a single simple hierarchy [43,44,45,46]. This is not automatically a disadvantage. A non-human repertoire can reveal useful antigenic vulnerabilities, but interpretation should be anchored in functional readouts such as neutralization, FcγR binding and protection in the relevant tissue. For example, porcine influenza-specific monoclonal antibodies were shown to recognize major haemagglutinin epitopes also targeted by humans, supporting the value of pigs for selected influenza antibody questions. Whereas HCDR3 length and composition primarily shape how an antibody recognizes and accesses antigenic epitopes, the IgG subclass and hinge region determine how effectively that binding event is converted into downstream effector functions (Figure 3).

## 7. IgG Subclass and Hinge Length

The hinge region connects the Fab and Fc domains and influences antibody flexibility. Longer and more flexible hinges, such as those found in IgG1 and IgG3 compared with IgG2 and IgG4, can improve access to antigens, complement proteins or Fc receptors and thereby influence effector function [22,47]. Subclass selection also matters because IgG1 and IgG3 generally bind activating FcγRs more efficiently than IgG2 or IgG4, making them more potent mediators of ADCC, ADCP and complement activation in many settings [27,28].

Recent hinge-engineering studies reinforce this point. IgG1 antibodies carrying IgG3-like hinge designs showed enhanced Fc-mediated phagocytosis in anti-streptococcal and SARS-CoV-2 antibody systems, illustrating that hinge geometry can change functional output even when antigen binding is not the only driver of activity [48].

Vaccine platform, dose interval, route of immunization, adjuvant and antigen persistence can all shape subclass distribution and Fc glycosylation through differences in innate sensing, tissue context and T follicular helper-cell support [49,50]. This matters for translation because subclass distributions and Fc receptor systems differ between species. A response that appears strongly Fc-functional in one animal may not have the same balance of activating and inhibitory Fc receptor engagement in humans unless the relevant species differences are accounted for during study design and analysis [29,30].

## 8. Fc Glycosylation

Fc glycosylation is another determinant of antibody efficacy. The conserved N-linked glycan in the IgG Fc region can alter antibody conformation and change the interaction between IgG and FcγRs or complement. Core fucosylation, galactosylation, sialylation and bisecting N-acetylglucosamine can all influence effector functions, including ADCC, ADCP and complement activation [38,51,52,53].

**Figure 4:**
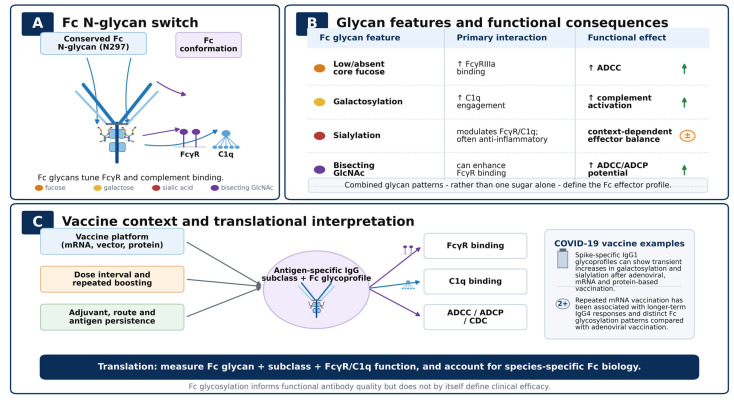
Fc glycosylation tunes antibody effector function and translational interpretation. (**A**) The conserved N-linked glycan at N297 in the IgG Fc region influences Fc conformation and modulates interactions with Fcγ receptors and C1q. Blue arrows indicate glycan-dependent effects on FcγR and C1q binding, whereas purple arrows indicate associated changes in Fc conformation. Colored circles represent fucose, galactose, sialic acid, and bisecting N-acetylglucosamine (GlcNAc). (**B**) Low or absent core fucose enhances FcγRIIIa binding and ADCC; galactosylation promotes C1q engagement and complement activation; sialylation modulates FcγR and C1q interactions in a context-dependent manner; and bisecting GlcNAc can enhance FcγR binding and ADCC/ADCP potential. Green upward arrows indicate enhanced activity, whereas the orange ± symbol indicates a context-dependent functional effect. Combined glycan patterns, rather than any single monosaccharide, define the overall Fc-effector profile. (**C**) Gray arrows converging on the central antibody indicate that vaccine platform, dose interval and repeated boosting, and adjuvant, administration route, and antigen persistence shape the antigen-specific IgG-subclass distribution and Fc glycoprofile. Colored arrows extending from the central antibody indicate the resulting functional measurements: the upper purple arrow denotes FcγR binding, the blue arrow denotes C1q binding, and the lower purple arrow denotes downstream ADCC, ADCP, and CDC readouts. COVID-19 vaccine studies illustrate transient changes in spike-specific IgG1 galactosylation and sialylation across vaccine platforms, as well as distinct longer-term IgG4 and Fc-glycosylation patterns following repeated mRNA vaccination. Translational interpretation should therefore measure Fc glycans together with IgG subclass, FcγR and C1q binding, and effector function while accounting for species-specific Fc biology. Figure generated with ChatGPT, version 5.5.

Recent COVID-19 vaccine studies show that antigen-specific IgG Fc glycosylation is dynamic and platform-dependent. Comparative analyses of spike-specific IgG1 glycoprofiles found transient increases in galactosylation and sialylation after adenoviral, mRNA and protein-based SARS-CoV-2 vaccination [54]. Other work showed that repeated mRNA vaccination could be associated with increasing longer-term IgG4 responses and distinct Fc glycosylation patterns compared with adenoviral vaccination [55]. These findings do not by themselves define clinical efficacy, but they show why Fc glycosylation and subclass should be considered when animal studies are used to predict human antibody function. These Fc-glycan-dependent effects are summarized in Figure 4, which links specific Fc glycan features to FcγR and complement engagement, downstream effector functions and translational interpretation in animal models.

## 9. Antibody Persistence and Immune Memory

Antibody half-life influences the duration of protection. Longer persistence at protective concentrations can extend the period during which antibodies neutralize pathogens, clear infected cells or block toxins [56]. IgG persistence is partly regulated by the neonatal Fc receptor (FcRn), which rescues IgG from intracellular degradation and thereby contributes to systemic antibody durability [57]. However, antibody half-life is only one part of vaccine-induced durability. Memory B cells and T cells can support a faster and broader response after re-exposure, and immune memory can matter even when circulating antibody titers decline [20].

For animal studies, this means that a single terminal titer is rarely sufficient. Time-course sampling, memory B cell analysis, durability of neutralization, Fc-mediated function and challenge timing can all change the interpretation of protection. These readouts are especially important when vaccines are intended to protect populations in which immune memory may be weaker or altered, such as young infants, older adults or immunocompromised individuals.

## 10. Why Not All Vaccine Candidates Succeed

The initial research phase of vaccine development often lasts 1 to 5 years, although pandemic programs may move faster when platform knowledge, manufacturing capacity and global coordination are already in place. During discovery, different approaches are explored and data are generated to support the design of a candidate. The preclinical and clinical phases then test whether the candidate is safe, immunogenic and effective enough for its intended use. Many candidates still fail before phase 3 or during phase 3 because the immune response is insufficient, the pathogen changes, the clinical endpoint is too difficult to demonstrate, or the safety profile is not acceptable [12,13,15].

## 11. Safety Concerns

Safety is the first requirement for any vaccine candidate. Toxicity concerns can emerge during phase 2 or phase 3 trials and may lead to discontinuation or non-approval. Such failures are relatively uncommon because vaccines undergo preclinical testing and earlier clinical phases before large efficacy trials. Nevertheless, safety evaluation must include the possibility of immune-mediated pathology, especially for respiratory viruses where vaccine-associated enhanced disease has been a historical concern [58].

## 12. Insufficient Efficacy

A vaccine that does not generate a strong enough immune response, or induces the wrong type of immune response, may fail to meet efficacy thresholds. Efficacy can be affected by antigen selection, adjuvant choice, dosing schedule, route of immunization, population age, pre-existing immunity, mucosal immune coverage and the complexity of the target pathogen. The increasing use of correlates of protection can help de-risk development, but unvalidated correlates can also mislead development decisions if the selected marker does not represent the biology of protection for the clinical endpoint [10,13].

## 13. Variability of the Pathogen

Some pathogens, including influenza virus, RSV and SARS-CoV-2, show antigenic variability. A vaccine designed against one strain or variant may provide limited protection against others. This can reduce overall efficacy even when the vaccine elicits strong immune responses to the original antigen. In such cases, animal studies should test not only homologous challenge, but also breadth against relevant variants or strains where suitable challenge models exist [23,37].

## 14. Inadequate Study Design

Study design is rarely the only reason a vaccine fails, but it can determine whether efficacy and safety are measured reliably. Inadequate sample size, the absence of suitable controls, inappropriate challenge dose or route, non-representative animal age, and readouts that do not correspond to the human endpoint can all reduce translational value. For preclinical studies, the key question is not whether an animal can be protected, but whether the model tests the mechanism and endpoint that matter in humans [11,59].

Translational failures can also reflect poor hypothesis definition, irreproducible data, ambiguous preclinical models, statistical weaknesses or limited transparency. For this reason, preclinical vaccine packages should define stage-gate criteria before study initiation, including which safety, immunogenicity or efficacy signals are sufficient to advance, modify or stop a candidate [15,60].

## 15. Regulatory and Manufacturing Constraints

Some candidates fail or stall because the product cannot be manufactured reproducibly at scale, because release assays and potency tests do not adequately reflect the mechanism of action, or because formulation changes cannot be bridged convincingly to earlier efficacy or immunogenicity data. Preclinical work should therefore not be isolated from chemistry, manufacturing and controls: the antigen, adjuvant, dose and delivery system tested in animals should be as close as possible to the product intended for clinical development [15]. Key takeaway: vaccine failure usually reflects a mismatch between mechanism, product design, endpoint and population rather than a single weak assay. Preclinical packages should therefore define go/no-go criteria before animal studies begin.

## 16. Designing Animal Studies to Improve Prediction in Humans

Humans and animals share many physiological and anatomical features. They have comparable organs and organ systems, and these systems often perform similar functions [59]. This similarity explains why animal models can be useful. At the same time, differences in natural infection, pathophysiology, immune cell biology, antibody repertoires, Fc receptor systems, innate sensing thresholds, glycosylation and immune experience can change how an animal responds to a pathogen or vaccine [28,61,62]. These differences should be treated as design variables, not as afterthoughts.

Predictive animal studies begin with the intended human question. If the clinical goal is to prevent infection, the model should support relevant exposure, replication and measurements of infection. If the goal is to prevent severe lower respiratory disease, the model should reproduce the target tissue and pathology. If the candidate is expected to work through Fc-mediated antibody functions, the model should allow meaningful interpretation of FcγR biology, glycosylation and effector cell activity.

To make model selection actionable, animal endpoints should be labeled by their relationship to human outcomes. Human-counterpart endpoints are measured similarly in animals and humans, such as neutralizing antibody titers, antigen-specific binding antibodies or T cell responses using aligned assays. Surrogate endpoints are not direct clinical outcomes but have a plausible mechanistic link to protection, such as lung viral load, nasal shedding, tissue pathology or transmission after challenge. Comparative or mechanistic endpoints are useful for ranking candidates or understanding biology but should not be treated as clinical correlates unless validated; examples include mouse weight loss, hypothermia, local cytokine patterns or model-specific histopathology [10,13,15,59].

A practical selection framework should combine species susceptibility, human-like clinical pathology, route of exposure and vaccination, age, sex, immune experience, immune mechanism, reagent availability, cost, throughput and ethical constraints. The most complex species is not automatically the best model; a well-powered and well-reported study in a simpler model may be more informative than a small, undercontrolled study in a closer but less tractable species. Study design and reporting should follow good animal research practice, including randomization, blinding where feasible, prespecified endpoints and transparent reporting of exclusions and sample size rationale [59,63].

Ethical model selection should include an explicit 3Rs rationale. Animals should be used only when no adequate non-animal method can answer the question, studies should be designed to reduce animal numbers without compromising inference, and procedures should be refined to minimize pain or distress. New approach methods can generate data before animal studies, but their ability to replace animal data depends on qualification and regulatory acceptance for the specific endpoint [64].

Target population relevance is particularly important for maternal, neonatal and pediatric vaccines. Pregnant people and children have often been underrepresented in vaccine development, so animal model data should be linked where possible to human cohort studies, natural history data and immunobridging strategies that reflect the intended perinatal or infant endpoint [65].

For high-consequence zoonotic viruses, translational model choice can be constrained by containment requirements as much as by biology. Biosafety level 2 (BSL-2)-compatible surrogate infection systems, minigenomes or virus-like-particle assays can support early candidate screening and mechanism studies, but confirmatory efficacy and safety claims still require authentic virus models under the appropriate containment conditions [66].

Immune experience is a specific example of this principle. Standard specific-pathogen-free rodents are valuable for controlled mechanistic studies, but they often have less microbial and antigenic exposure than adult humans. Co-housed or so-called dirty mouse approaches can produce immune phenotypes closer to adult human immunity and may alter vaccine responses; however, exposure method, microbiome composition and housing history should be reported and standardized before such data are used for translation [67,68,69]. Key takeaway: Translational relevance is endpoint-specific. A model can be appropriate for dose selection or mechanism, but inappropriate for transmission, severe disease or Fc-mediated biology unless those endpoints are explicitly represented and measured.

## 17. Match the Model to the Biology of Infection

Influenza virus illustrates how infection biology can determine model value. Mice are not naturally susceptible to many human influenza strains, so experimental studies often use mouse-adapted viruses. These infections can begin in both upper and lower respiratory tracts and may produce pneumonia, hypothermia and hypoxia rather than the fever and upper respiratory presentation typical of many human influenza infections [70,71]. Mice are therefore useful for mechanism and genetics, but they are not always the best model for transmission or human-like clinical disease.

Ferrets provide a closer model for several aspects of human influenza. They can be infected with primary human influenza strains, develop upper respiratory tract disease, show clinical signs resembling those observed in humans and transmit respiratory viruses efficiently [72,73]. For influenza vaccine studies, this makes ferrets useful for assessing vaccine-induced effects on viral shedding, transmission and upper airway infection.

SARS-CoV-2 showed the same principle during the COVID-19 pandemic. Hamsters, ferrets, mice and non-human primates were all useful, but for different questions [74]. Golden Syrian hamsters reproduced several features of mild human infection and became useful for pathogenesis and countermeasure studies [75,76]. Ferrets were useful for transmission studies [73,77]. Non-human primates supported evaluation of lower airway disease, viral replication and immunogenicity for vaccine candidates, including mRNA-1273 and ChAdOx1 nCoV-19 [78].

Model matching should also include severe or extra-respiratory endpoints. When vaccines are intended to prevent complications such as influenza-associated encephalitis or encephalopathy, respiratory viral load and systemic antibody titers are insufficient; models and complementary systems should capture the relevant tissue pathology, neuroinflammatory features or barrier dysfunction where feasible [79].

## 18. Account for Species-Specific Antibody Biology

Species-specific antibody features are important when selecting and interpreting animal models. Differences in antibody glycosylation, Fc receptor biology, HCDR3 distribution, hinge structure and subclass composition can influence neutralization, CDC, ADCC and ADCP. These differences do not make an animal model unusable, but they determine which conclusions can be drawn directly and which require bridging assays.

For Fc-mediated questions, model selection should include the Fc receptor system. Mouse FcγRs are not identical to human FcγRs, and the balance of activating and inhibitory receptor engagement may differ between species [27,29]. Humanized mouse models that express human FcγRs or antibody constant regions can help address specific Fc-function questions, but their glycosylation machinery, immune cell compartments and infection biology may still remain partly murine [80].

For antibody repertoire questions, model choice should consider whether the species can generate antibody classes and paratopes relevant to the human target. Rabbits can generate useful high-affinity antibodies and have repertoire features that differ from humans and mice [44]. Ferrets are central for influenza and other respiratory virus studies, but historically have had fewer immunological reagents and less complete immune-repertoire annotation than mice or humans [46]. Pigs and mini pigs offer anatomical and immunological similarities to humans and may be valuable for selected respiratory, mucosal and antibody discovery questions [81].

## 19. Choose Readouts That Can Bridge Animal and Human Data

A model becomes more predictive when animal and human data share interpretable readouts. Neutralization assays, binding antibody titers, FcγR binding, ADCC, ADCP, complement activation, tissue viral load, pathology and durability can all be useful, but the right set depends on the vaccine mechanism and clinical endpoint. For respiratory vaccines aimed at limiting spread, shedding, contact or aerosol transmission and, where feasible, airborne-virus or environmental sampling should be aligned with the transmission endpoint [82]. Using standardized assays across animal and human samples can improve bridging, especially when the same antigen, assay controls and reporting units are used.

Recent correlate-of-protection studies show the value of such bridging. Neutralizing and binding antibody levels were linked to clinical efficacy in major SARS-CoV-2 vaccine trials [24,25]. At the same time, studies of Fc-mediated functions show that protection can depend on antibody quality and tissue context, especially when neutralization is weakened by antigenic change [35,36,37,50]. These findings argue for preclinical designs that measure several immune dimensions rather than relying on a single terminal titer.

## 20. Use Integrated Immunogenicity Analysis

In vitro and ex vivo assays can support animal-to-human bridging when they are selected for the expected mechanism of action. A minimal package often includes binding antibody assays, neutralization assays, T cell activation or cytokine assays, and durability measurements. When Fc-mediated protection is plausible, FcγR binding, ADCC, ADCP, CDC/complement activation, IgG subclass and Fc glycosylation should be measured rather than inferred from total IgG alone. Human-relevant new approach methods, including air–liquid interface cultures, precision-cut tissue slices, organoids, 3D bioprinted tissues, organ-on-chip systems and computational models, can refine mechanistic screening, support the 3Rs and prioritize candidates before in vivo challenge studies; however, they cannot yet replace whole-organism assessment of safety, biodistribution, tissue tropism, shedding, transmission or durability [49,83,84,85,86].

Systems serology and multiplex immunoassays can combine these variables into signatures that compare vaccine platforms, adjuvants and schedules. Such analyses should be standardized with shared controls, dynamic ranges and prespecified statistical models, then validated across time points and, where possible, across species or early human cohorts. This approach helps identify which animal signals are mechanistically relevant and which are species-specific artifacts [15,49,50].

High-dimensional omics and AI/ML tools can help identify immune signatures, prioritize candidates and support interim go/no-go decisions, but their use should be deliberately cautious. Preclinical vaccine datasets are often small, heterogeneous and vulnerable to batch effects, overfitting, data leakage and platform-specific signatures that do not generalize. Predictive models should therefore be linked to prespecified biological hypotheses, trained with transparent feature selection, validated in independent cohorts and interpreted as decision-support tools rather than stand-alone evidence of efficacy [14,15].

## 21. Model-Specific Considerations

Common vaccine models are best viewed as complementary tools rather than as a hierarchy. Selection should be driven by the pathogen, vaccine platform, intended route of administration, expected immune mechanism and clinical endpoint. These mechanistic considerations should guide animal model selection. Because no single species captures all aspects of human infection, antibody biology and clinical disease, common models are best viewed as complementary systems whose value depends on the pathogen, vaccine platform, immune mechanism and endpoint under study (Table 1).

## 22. Translational Endpoint Alignment: Influenza and COVID-19 Examples

Influenza and COVID-19 illustrate how the same animal species can answer different questions at different development stages. Challenge studies should define the strain or variant, challenge dose, route, timing after vaccination and success criteria before the experiment begins; otherwise, protection in an animal challenge may not map onto the intended human endpoint. Representative stage-specific model selections and endpoint interpretations are summarized in Table 2.

The table classifies endpoints by their intended translational role. Counterpart biomarkers support direct animal–human comparison when assays are aligned; surrogate endpoints support inference about protection; comparative endpoints are useful for ranking or mechanism but require confirmatory bridging.

## 23. Conclusions

Viral vaccine development is most reliable when immune mechanism, model system, challenge design and biomarker interpretation are aligned from the outset. A candidate that protects an animal from experimental challenge has not necessarily shown that it will protect humans; the model must reproduce the relevant part of the human infection, support the intended mechanism of protection and generate readouts that can be compared with human data.

Humoral and cellular immunity should both be considered during this alignment. For antibody-focused candidates, study design should include the relevant neutralization, Fc receptor, subclass, glycosylation and persistence measurements. For candidates expected to prevent severe disease, control tissue infection, or rely on T cell activity, cellular immune readouts and tissue-specific endpoints are equally important.

No single model is sufficient for all vaccine development questions. A stronger strategy is a staged model cascade: tractable models for mechanism and dose selection, disease-relevant challenge models for pathophysiology or transmission, and standardized human-relevant assays for immunobridging. This approach can improve interpretation of preclinical data and reduce the risk of advancing vaccine candidates that are unlikely to succeed in clinical trials.

## Figures and Tables

**Table 1:** Common preclinical animal models for viral vaccine studies and the main design questions they help answer.

Model or Group	Useful When the Main Question is…	Key Translational Cautions
Mice and rats	Mechanism, genetics, early safety, dose selection and platform comparison.	Pathogens may require adaptation or transgenic susceptibility; SPF immune experience and murine Fc biology can limit translation [67,69].
Hamsters	Respiratory virus replication, pathology and countermeasure testing, especially for coronaviruses and selected influenza or RSV questions.	Fewer immunological reagents than mice; hamster antibody glycosylation and Fc-receptor biology require species-specific interpretation [75,76,85].
Cotton rats and guinea pigs	Selected respiratory virus models, including RSV and influenza; useful when permissiveness, lung pathology or respiratory physiology are central.	Limited genetic tools and sampling depth; immune kinetics and reagent availability can restrict mechanistic interpretation [15].
Ferrets	Influenza and other respiratory virus pathogenesis, upper airway infection, shedding and transmission.	Lower throughput and fewer immune reagents than mice; best suited to respiratory questions rather than broad immunological screening [72,73].
Rabbits	Immunogenicity, toxin studies, repeated sampling and antibody discovery.	Repertoire generation, glycosylation and Fc interactions differ from humans, so functional bridging is needed [44].
Pigs and mini pigs	Body size, skin, mucosa, respiratory physiology, adjuvant benchmarking and immunosafety.	Higher cost and facility demands; porcine FcγR binding and IgG glycosylation should be measured when Fc function drives translation [81,87,88].
Non-human primates	Late-stage bridging, complex immunology and pathogens where no smaller model captures the relevant biology.	Use must be justified by a specific translational question because ethical, regulatory, availability and cost constraints are substantial [7,78,89].

This table is a starting point, not a ranking. Strong preclinical packages often combine a tractable model for mechanism and dose selection, a disease-relevant model for challenge or pathophysiology, and standardized assays or early human immunoprofiling for bridging.

**Table 2:** Examples of stage-specific model selection and endpoint interpretation for viral vaccine studies.

Use Case	Preferred Model(s)	Endpoints and Biomarker Class	Interpretation Criteria
Influenza: early mechanism and dose selection	Mice or rats, with confirmatory respiratory models when needed	Aligned neutralization or HAI-like assays and binding antibodies are human-counterpart readouts; weight loss, survival and lung viral load after challenge are comparative or surrogate endpoints.	Use for candidate ranking, mechanism and dose selection. Do not infer human transmission or clinical disease protection from mouse endpoints alone.
Influenza: upper airway disease and transmission	Ferrets	Nasal shedding, upper airway viral load, clinical signs and contact or aerosol transmission are surrogate endpoints linked to human respiratory spread.	Interpret strongest when challenge virus, exposure route and transmission set-up resemble the intended human question, including homologous and relevant heterologous strains.
COVID-19: pathogenesis and countermeasure screening	Hamsters or susceptible/transgenic mice	Weight change, lung viral load, histopathology and neutralization after challenge provide surrogate or comparative evidence of protection.	Useful for rapid ranking and pathology; limited reagent depth and species-specific disease patterns require bridging with standardized immune assays.
COVID-19: transmission and upper airway replication	Ferrets	Nasal viral shedding and animal-to-animal transmission are surrogate endpoints for spread; clinical disease is usually limited.	Best suited to transmission questions, not severe lower-airway disease or full systemic immunology.
COVID-19: late-stage bridging and lower-airway protection	Non-human primates	Neutralization, binding antibodies, T cell responses and Fc readouts are human-counterpart immune markers; bronchoalveolar, nasal and lung viral loads and pathology are challenge surrogates.	Most informative when the same assays, antigen panels and reporting units are used in animals and clinical samples; controlled challenge remains a surrogate for field efficacy.

## Data Availability

No new data were created or analyzed in this study. Data sharing is not applicable to this article.
